# Image and Diagnosis Quality of X-Ray Image Transmission via Cell Phone Camera: A Project Study Evaluating Quality and Reliability

**DOI:** 10.1371/journal.pone.0043402

**Published:** 2012-10-17

**Authors:** Hans Goost, Johannes Witten, Andreas Heck, Dariusch R. Hadizadeh, Oliver Weber, Ingo Gräff, Christof Burger, Mareen Montag, Felix Koerfer, Koroush Kabir

**Affiliations:** 1 Department of Orthopedic and Trauma Surgery, University Hospital Bonn, Bonn, Germany; 2 Department of Radiology, University Hospital Bonn, Bonn, Germany; 3 Department of Anesthesiology and Intensive Care Medicine, University Hospital Bonn, Bonn, Germany; The University of Queensland, Australia

## Abstract

**Introduction:**

Developments in telemedicine have not produced any relevant benefits for orthopedics and trauma surgery to date. For the present project study, several parameters were examined during assessment of x-ray images, which had been photographed and transmitted via cell phone.

**Materials and Methods:**

A total of 100 x-ray images of various body regions were photographed with a Nokia cell phone and transmitted via email or MMS. Next, the transmitted photographs were reviewed on a laptop computer by five medical specialists and assessed regarding quality and diagnosis.

**Results:**

Due to their poor quality, the transmitted MMS images could not be evaluated and this path of transmission was therefore excluded. Mean size of transmitted x-ray email images was 394 kB (range: 265–590 kB, SD ±59), average transmission time was 3.29 min ±8 (CI 95%: 1.7–4.9). Applying a score from 1–10 (very poor - excellent), mean image quality was 5.8. In 83.2±4% (mean value ± SD) of cases (median 82; 80–89%), there was agreement between final diagnosis and assessment by the five medical experts who had received the images. However, there was a markedly low concurrence ratio in the thoracic area and in pediatric injuries.

**Discussion:**

While the rate of accurate diagnosis and indication for surgery was high with a concurrence ratio of 83%, considerable differences existed between the assessed regions, with lowest values for thoracic images. Teleradiology is a cost-effective, rapid method which can be applied wherever wireless cell phone reception is available. In our opinion, this method is in principle suitable for clinical use, enabling the physician on duty to agree on appropriate measures with colleagues located elsewhere via x-ray image transmission on a cell phone.

## Introduction

Telemedicine and teleradiology, in particular, are of increasing importance in daily clinical practice. Teleradiology is a highly evaluated and widely used method despite its high costs and technical complexity [1], [2]. In the surgical field, no additional benefits from teleradiology have resulted due to existing financial, technical and legal obstacles (Tables 1, 2). Thus, teleradiology remains mostly localized and personal (radiology specialist).

**Table 1:** Teleradiology requirements.

1.	Adequate technical resources at place of examination/evaluation
2.	Personnel skilled in technical equipment present at place of examination
3.	Rapid and reliable transmission
4.	Assessment by medical specialist at reception point

**Table 2:** Uses of teleradiology.

1.	Emergency teleradiology (emergency consulting)
2.	Second opinion (teleconsulting)
3.	Joint discussion on findings (teleconference)
4.	Distribution of images and findings (e.g. to referring physician)
5.	Scientific cooperation
6.	Linking with home offices (e.g. background duty radiology)

Consequently, there is no consistent further development or opening of telemedicine for telesurgery or telemetric orthopedic and trauma surgery, while especially in orthopedic and trauma surgery, a rapid and easy to use system for joint evaluation of x-ray images in an emergency would be desirable. Such a system could enable valid assessment of x-ray images independent of location.

A teletrauma unit could enable on-call services to establish diagnosis and/or indication for surgery more rapidly and thus speed up patient treatment in an emergency. Especially during on-call duties, situations may arise whereby medical assistants who are not yet able to judge complex situations must consult with more qualified specialists on background duty. However, in complicated cases, even the specialist on background duty has to occasionally consult with experienced colleagues or the head of the department. In these joint consultations, x-ray images are usually verbally described more or less accurately.

In view of the development of ubiquitously available cell phones and cell phone networks, telemedicine has already expanded towards a Global System for Mobile Communications-based telemedicine [3]. Ricci could show that decision making regarding indication for surgery is possible in joint assessment by telephone following electronic transmission of x-ray images via GSM phone [4]. Whether transmission of x-ray images of various anatomical regions and injuries is of sufficient quality and reliability has not been studied to date. However, transmission of photographed x-ray images via email or cell phone has certainly become a regular practice in many clinics today.

In the present project study, we examined whether transmission time, quantity and quality of data of transmitted x-ray images of various anatomical regions and their typical injuries are sufficient to enable rapid and safe assessment of these images.

## Materials and Methods

### 1. Image material

In total, 100 x-ray images of nine different body regions were selected for this study. Included were 10 images of pediatric injuries and 30 images without pathological findings. All images were selected within a two-week period from the accident and emergency department and given final diagnoses after being read in person by the author H.G. On the selected images with pathological findings, the findings were evident with no hidden cracks or hard to find fracture lines. A graded classification of pathological findings was not applied; the final diagnosis just contained positive or negative pathological finding. These x-ray images were then photographed and sent out via email to the reviewers. All the diagnoses determined then by the remote reviews were ultimately compared to these actual final diagnoses.

### 2. Photography of the image material

We used a Nokia N 95 (Picture S1, Nokia Oy, Espoo, Finland) cell phone with integrated digital camera and an additional SD card, where up to 8 gigabyte of photographs and other data can be stored. Resolution is up to 5 megapixels due to an aspect ratio of 4∶3 and 2592×1944 pixels (5,038,848 pixels) according to the product specification. Included are integrated flash, auto focus and black/white as well as color mode. Focal length of optics is 5.6 mm, corresponding to a focal length of 35 mm, thus comparable to commercially available 35 mm cameras. Focus range is between 10 cm and infinity. Photographs can be stored as JPEG or exchangeable image file format (EXIF).

To ensure best possible quality, the photographs were taken in a darkened room with full focus on the x-ray image as displayed on the screen. Flash was deactivated. Photographs were taken in the black/white mode to avoid any green casting. Distance between camera and object was 30 cm. Each image was photographed several times with the highest possible resolution and the best photographs were selected and numbered consecutively. Photographs were stored as JPEG and transmitted via MMS (multimedia message service) and email.

Further image quality measurements such as contrast resolution and contrast-to-noise ratio were not assesed.

### 3. Transmission and reception of images

The consecutively numbered graphic data were sent immediately after photographing to the five reviewers as email attachments. Additionally, a copy was sent to a control email address to document correct transmission and transmission time. In a second step, transmission via MMS was done in the same manner.

### 4. Assessment of images

Assessment of transmitted x-ray images was carried out on a laptop computer (Lenovo T500, screen resolution 1280×800, 32-bit color depth) regarding quality, visible pathology and indication for surgery. On a scale ranging from 1 (very poor) to 10 (excellent), image quality was arranged regarding sharpness, brightness and contrast. Pathology and/or indication for surgery were assessed by naming the main or obvious pathological changes, e.g. “fracture of the medial femoral neck”, “pneumothorax” or “fracture of the anterior pelvic ring”. In the absence of pathological findings, assessment resulted in “normal finding”.

The data were transmitted via email or MMS, respectively, to five medical specialists for assessment.

The five reviewers were:

1. Medical director, specialist at the Department of Orthopedic and Trauma Surgery
2. Senior physician, specialist at the Department of Orthopedic and Trauma Surgery
3. Specialist at the Department of Orthopedic and Trauma Surgery
4. Specialist at the Department of Orthopedic and Trauma Surgery
5. Senior physician, specialist at the Department of Radiology

### 5. Ethics and data protection

Following consultation with the data protection officer appointed by the University Hospial and the local ethics committee, all patient data on the x-ray images (name, first name, date of birth and patient identification number) were blinded prior to photographing. The five reviewers received no information on the patients. None of the pictures were shown to a physician outside our hospital. Therefore, according to prior agreement with the local ethics committee, verbal or written informed consent was not obtained.

### 6. Evaluation

All data were entered into an Excel file according to parameter, parameter characteristic or parameter value, respectively. Summing up and statistical calculations were carried out with the BiAS program (Biometric analysis of samples, Ackermann, 1989–2001). Mean value, standard deviation, median with maximum and minimum values and confidence interval (CI) of 95% were calculated. For comparative assessment, the Wilcoxon-U-test for non-parametric values was applied. A box plot was used to illustrate the calculated values. The relationship between cumulative values and image quality and concurrence ratio of diagnosis was calculated and illustrated as single linear correlation (Pearson).

## Results

In total, 100 blinded x-ray images were separately sent to the individual reviewers.

Selection of diagnoses/body locations had been carried out prior to transmission based on clinical and radiological findings.

As the quality of the images transmitted via MMS was evidently very poor these were rejected for assessment by all reviewers. Therefore, any assessment of these images was discontinued due to obvious quality defects.

Mean size of images transmitted via email was 394 kB (range: 265–590 kB, SD ±59). Mean value of transmission time was calculated as 3.29±8 min (CI 95%: 1.7–4.9) (median: 0; 0–37).

Table 3 shows the distribution of the final findings. In 30% of images, no pathological or clinically relevant findings were present (normal findings), while 10% of images showed pediatric injuries. The remaining images depicted typical injuries in various body locations.

**Table 3:** Distribution of diagnoses based on clinical and radiological findings.

diagnosis/body location	frequency	share (%)
1. no findings	30	30
2. spinal column	4	4
3. thorax	6	6
4. shoulder and upper arm	12	12
5. elbow and lower arm	6	6
6. hand	5	5
7. pelvis/hip	8	8
8. femur and knee	7	7
9. lower leg and upper ankle joint	8	8
10. foot	4	4
11. pediatric injuries	10	10

**Figure 1:**
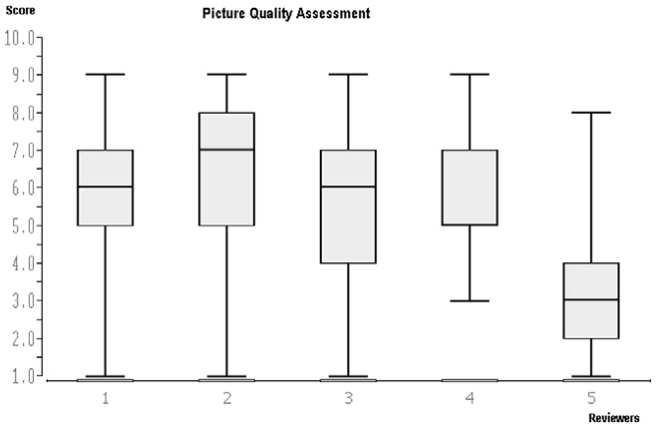
Box plot with median, Q1 and Q3, minimum and maximum values for assessment of image quality by five specialists (score 1–9). Comparison between reviewers 1–4 and reviewer 5: p<0.01.

The four orthopedic and trauma specialists assessed the image quality (score 1–10) with mean values between 5.4 and 6.2 (mean value: 5.8, median: 6) (Table 4, Fig. 1) without significant differences between the assessments of the individual reviewers, whereas the fifth member of the panel, a specialist in radiology, assessed the quality of the images with a lower mean value of 3.1. Hence, his assessment differed significantly from those of his colleagues. These differences became also noticeable when summing up the individual image assessments (Table 5), whereby the mean value of the sum of the assessments by specialists 1–4 was significantly different (p<0.01) compared to the assessments of the radiology specialist. No correlation was found between final diagnoses and assessments of image quality (correlation coefficient: −0.15; p>0.1).

**Table 4:** Assessment of image quality (score: 1–10) after transmission.

reviewer	MV ± SD	CI: 95%
1	6.1±2	5.7–6.4
2	6.2±2	5.9–6.6
3	5.4±2	5.0–5.7
4	5.7±2	5.4–6.0
5	3.1±1	2.8–3.4
reviewers 1–4 vs. reviewer 5: p<0.01		

Mean values (MV), standard deviations (SD) and confidence intervals (CI: 95%) from statements of individuals reviewers.

Significant differences were found between values from specialists 1–4 and values from specialist 5 (radiology).

**Table 5:** Assessment of image quality (score 1–10) by four specialists in orthopedics/surgery/trauma surgery and one specialists in radiology.

assessment score	5 reviewers sum n (%)	reviewers 1–4 sum/4 n = %	reviewer 5 sum n = %
1	18 (3.6)	1.5	12.0
2	36 (7.2)	2.0	27.0
3	59 (11.8)	8.5	25.0
4	59 (11.8)	10.0	20.0
5	80 (16.0)	18.0	8.0
6	87 (17.4)	20.0	6.0
7	88 (17.6)	22.0	1.0
8	54 (10.8)	13.0	1.0
9	19 (3.8)	5.0	0
sum total	500	100	100
mean value	5.1	5.8	3.1

Left column: all reviewers; middle column: orthopedics/surgery/trauma surgery specialists; right column: radiology specialist; median of difference: reviewers 1 to 4 vs. reviewer 5 = 2.75 (p<0.01).

Comparison between the final diagnoses and the statements issued by the five reviewers of the transmitted images resulted in confirmed findings in 83.2±4% of cases (mean value ± SD; median 82; 80–89%). Table 6 shows the percentage shares of confirmed findings, whereby a distinction was made between statements issued on all findings and statements issued on normal findings or findings with a final diagnosis, respectively. No significant difference was found regarding concurrence ratio. An additional evaluation showed that, on average, accurate diagnosis was reached unanimously by all specialists in 76% of all transmitted images (Table 7), while in 7% (median), statements regarding diagnosis were unanimously false, and in 18%, concurrence ratio differed between the individual reviewers.

**Table 6:** Tally of accurate confirmations of final diagnoses after assessment of transmitted images by 5 specialists.

confirmation of diagnosis by 5 specialists	all x-ray images	x-ray images without given diagnosis no findings	x-ray images with given diagnosis
assessment total	n = %	n (%)	n (%)
0 accurate	3	1 (3)	2 (3)
1 accurate	3	1 (3)	2 (3)
2 accurate	6	1 (3)	5 (7)
3 accurate	12	5 (17)	7 (10)
4 accurate	16	5 (17)	10 (14)
5 accurate	61	17 (57)	44 (63)
total	100	30	70

No significant differences were found regarding the specifications “no pathological findings“ and “with diagnoses”.

**Table 7:** Comparison of diagnosis concurrence ratio based on individual reviewers.

reviewer	false - false	false - accurate	accurate - false	accurate – accurate
1 vs. 2	18	0	2	80
1 vs. 3	8	7	12	85
1 vs. 4	7	4	13	76
1 vs. 5	8	12	12	68
2 vs. 3	7	8	11	74
2 vs. 4	6	5	12	82
2 vs. 5	7	13	11	69
3 vs. 4	5	6	10	79
3 vs. 5	5	15	10	70
4 vs. 5	7	13	4	76
median	7	7.5	11	76
CI 95%	5–8	4–13	4–12	69–82

On average, a 76% total agreement of accurate diagnosis occurred.

Table 8 shows the percentage shares of accurate findings after image transmission issued by the specialists depending on the final diagnoses/body locations.

**Table 8:** Confirmation of correct remote diagnosis depending on selected diagnoses/body locations.

diagnoses/body location	confirmation	share (%)
no pathological findings	17/30	57
spinal column	3/4	75
thorax	1/6	17
shoulder and upper arm	10/12	83
elbow and lower arm	4/6	67
hand	3/5	60
pelvis/hip	7/8	88
femur and knee	6/7	86
lower leg and upper ankle joint	5/8	63
foot	2/4	50
pediatric injuries	3/10	30
total	61/100	61

**Figure 2:**
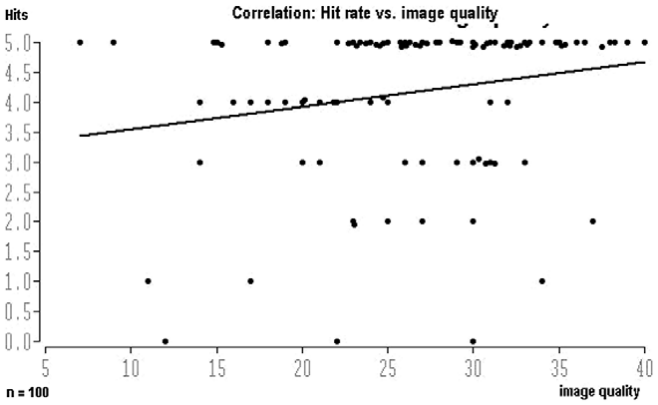
Diagram of correlation between sum of confirmed diagnoses by the five specialists (ordinate: 0 = false, 1 = accurate) and sum of assessments of image quality (abscissa: score 1–9). Correlation ratio: 0.204; exceeding probability: p = 0.04.

As the number of cases was in some parts low, direct conclusions can only be drawn with reservations. Of note is the low concurrence ratio in the thoracic region and in pediatric injuries. In the thoracic region, accurate diagnosis was stated by all reviewers in only one case, while in another case, all reviewer findings were false. The percentage of accurate diagnosis by all reviewers – excluding thoracic region and pediatric injuries – was 71%. Including the thoracic region and pediatric injuries only 61% were reached. Here it must be taken into account that the percentage share of accurate findings by the reviewers depended on image quality. Comparison between the sum of image quality assessments and the sum of accurate findings shows a direct correlation between both parameter values (Fig. 2) with a correlation coefficient of 0.204 (p<0.05).

During data transmission via cell phone email, the actual costs were calculated depending on data size. As each of the 100 photographs was sent to six recipients – five specialists and one control recipient – 600 emails were sent in total. The costs were 0.795 EUR per transmission.

## Discussion

The use of cell phones for medical purposes is not new. In fact, it has been evaluated several times in patient monitoring and patient communication [5], [6]. Uses range from monitoring of asthmatic patients via transmission of breath sounds, to management of diabetes outpatients, to transmission of photographs of the skin for diagnosis and therapy control in dermatological diseases [7], [8], [9]. Reponen et al. and Phabhal et al. were able to show in their studies that in the area of neurosurgery, transmission of individual CT (Computerized Tomography) images via cell phone for assessment of injuries is simple, easy and more than adequate [10], [11]. In-house solutions via cell phone or PDA (Personal Digital Assistant) for PACS (Picture Archiving and Communication System) are currently being tested in clinical practice [12].

In the present study, we tested whether transmission of x-ray images via email on a cell phone from the clinic to a laptop is a reliable. It is certainly not the best form of consultation since further verbal communication with additional description of the images did not take place. Under standard conditions (darkened room, x-ray alternator, black/white camera mode without flash) average size of email was 394 kB, due to compression to JPEG format as well as black/white mode. Major advantages include good storage facilities on the cell phone and rapid transmission and availability on a laptop or desktop computer. Apart from a few outliers, time between sending of the photographs and assessment on the desktop/laptop computer was less than 60 sec. Only in ten photographs did the transmission time exceed 10 min. Costs were very low at approximately 0.80 EUR per transmission.

Rate of accurate diagnosis and indications for surgery is high with a concurrence ratio of 83%. However, only in 61% of cases did all reviewers reach the accurate diagnosis unanimously. Also, depending on body location, considerable differences existed with a higher concurrence ratio in long tubular bones and large joints than in the spinal region and small joints, while photographs of thorax x-rays had the poorest results. Of note are also the low numbers of accurate diagnoses in cases without pathological findings and in pediatric injuries.

In x-ray images from the thoracic region, not only numerous bones, but also the subtle details of the lung must be assessed. Therefore, assessment of a normal x-ray image of the thoracic region is more difficult than assessment of a skeleton x-ray image. As the reviewers' assessment of image quality of the photographs of thoracic x-rays was slightly higher than the mean, poor image quality cannot be the cause of the lower accuracy of diagnosis. However, not only trauma images were included in the photographs but also assessments of pleural effusion or pulmonary congestions which are rarely required by trauma surgeons.

The moderately successful results in the assessment of pediatric injuries images is not surprising either as x-ray images of pediatric results are difficult to assess due to the as yet incomplete epiphyseal fusion. Also, the quality of the pediatric injury photographs was graded somewhat lower with an average rating of 4.4. Furthermore, since the photographs of the pediatric x-rays were presented last to the reviewers, fatigue may have played a role in the relatively low number of accurate diagnoses.

Despite the good results, this method should not be overrated. First, it must be stressed that only conventional x-ray images were viewed which obviously continue to play an important part in radiological examinations and are essential in many areas. However, in many orthopedic and trauma cases, multiple slice imaging via CT or MRI (Magnetic Resonance Imaging), which is superior to conventional x-ray imaging, is indicated. Also, it must be noted that while generally a significant correlation was found between concurrence ratio and image quality, the specialist in radiology assessed image quality significantly lower than the other medical specialists. Furthermore image quality was only assessed by subjective rating to keep the study easy and outside the requirements of teleradiology. In an established cell phone consulting system this aspect would be necessary according to quality management. Thus, right from the outset the transmitted image material should be assessed critically and if necessary, diagnosis must be stated with caution.

Another critical aspect of the assessment results is the discrepancy found between the concurrence ratios of the individual reviewers (Tab. 5). This is, inter alia, possibly due to the experimental nature of this study. In realistic circumstances, image transmission would be supplemented with verbal information regarding case history, record of clinical findings etc., thus possibly optimizing the concurrence ratio regarding diagnosis.

Although this is a reliable and thus functional method, the legal basis remains unclear. This is not a teleradiology method since the European X-ray Ordinance stipulates specific workstations for viewing and diagnostics. The method presented here cannot meet these high standards of quality. Diagnosis on a laptop would simply be illegal, with extensive consequences due to a potentially inadequate or omitted treatment for a disease based on a transmitted and inaccurately interpreted photograph.

However, these were not our aims. The images transmitted via email are only to serve as decision-making aids in joint assessment of findings or treatment plans by telephone, while detailed recording of the medical history and thorough physical examination of the patient remain absolutely essential. No therapy decision must be taken based on transmitted images alone. Indication for therapy must continue to be based on the patient. In was our intention to demonstrate the possibilities available with relatively simple technical means. It is a cost-effective and rapid method which can be applied almost anywhere.

In our opinion, this method is in principle suitable for clinical use enabling the on-duty physician in hospital to agree on further treatment with the background duty specialist by x-ray transmission via cell phone, thus possibly saving the background duty specialist from traveling to the hospital. This could result not only in increased patient comfort, but also in a reduced demand on the background duty specialist, saving time and hence improving compliance with the European Working Hours Act.

In a real clinical setting the transmitted images would be discussed additionally with the physician on duty or in future with photographs and notes from the electronic patient files.

## Conclusions

In this study, the photographing and mailing of x-ray images via cell phone has been proven to be a valid and applicable method for x-ray image evaluation. Necessary requirements include a cell phone camera of at least 5 megapixels and access to email transmission.

Our study confirms that the quality of a photographed and electronically transmitted x-ray image may be suitable in facilitating decision-making by the background duty specialist, possibly making the personal appearance of a background specialist in some cases unnecessary.

## Supporting Information

Picture S1 Nokia N95.
(TIF)
