# Supplementary figures and images for: Image and Diagnosis Quality of X-Ray Image Transmission via Cell Phone Camera: A Project Study Evaluating Quality and Reliability
Source: PLoS One. 2012 Oct 17;7(10):e43402. doi: 10.1371/journal.pone.0043402 (PMC3474770; doi:10.1371/journal.pone.0043402)

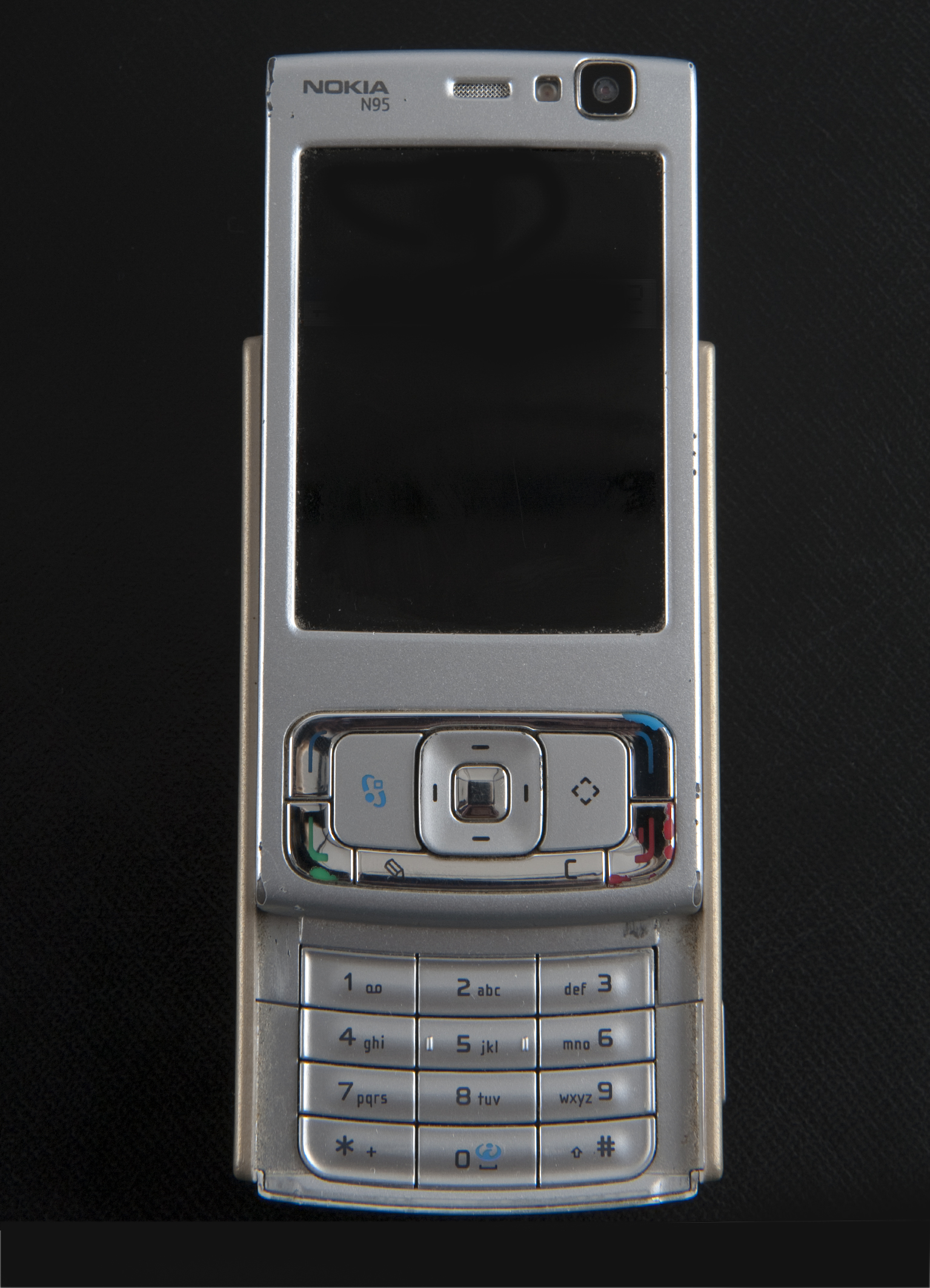

Supplement: Picture S1 — Nokia N95. (TIF) [file pone.0043402.s001.tif]
